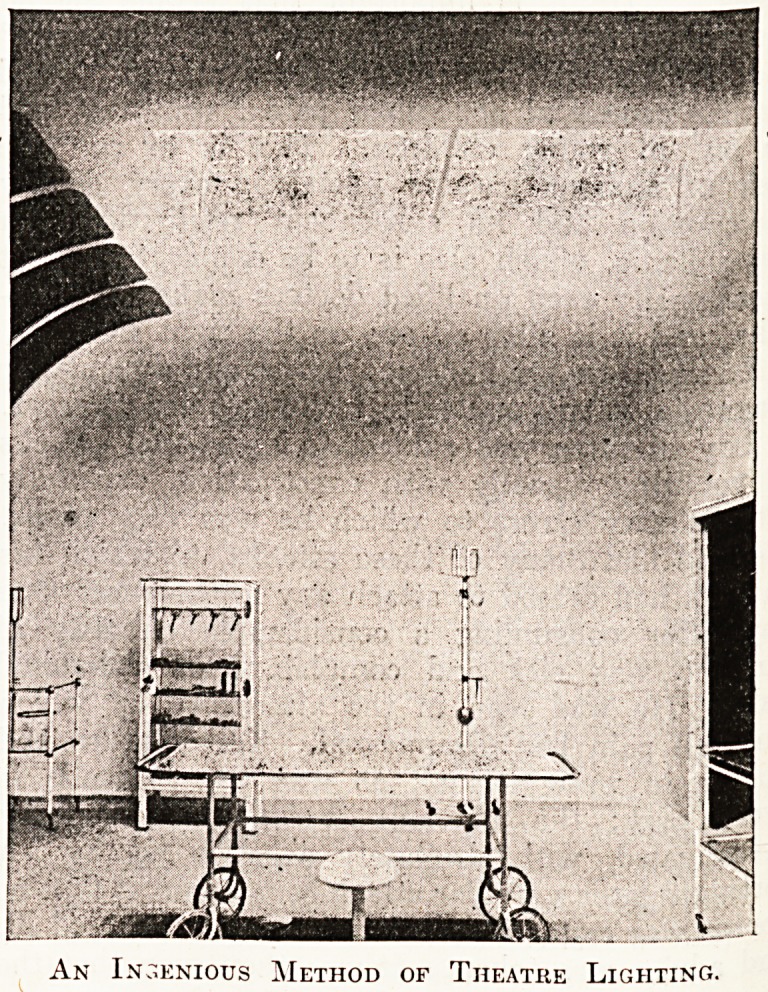# The Artificial Lighting of Hospital Wards

**Published:** 1913-05-03

**Authors:** 


### THE ARTIFICIAL LIGHTING OF HOSPITAL WARDS.

The indirect system of lighting a large room by screens which cut off all direct rays from the source of light to the eyes of those using the room and reflect a diffused light on to the walls or

"The Eye-Rest" Fitting.

ceiling has been in favour for a long time in picture galleries and similar places, and has lately been extensively adopted also in private houses. Hitherto it has not been taken up much for hospital-ward purposes; and this is surprising when the advantages of such a system for this kind of room are pondered.

Sick people, for one thing, are often intolerant of degrees of glare or strong light which in health they would enjoy. In bed in a ward, again, their gaze is much more directed towards the ceiling and upper part of the room than if they were sitting or standing; so that a pendent light may easily annoy them, when their attendants are unconscious of any discomfort from it. And, finally, they frequently spend long periods lying awake during the night in a ward where there must be some degree of illumination for the nurse-in-charge to do her duties by.

In the new Chiswick Cottage Hospital an attempt has been made by the architects, Messrs. Scott and Fraser, to take full advantage of all the most modern improvements in electric-lighting design; and, in conjunction with the British Thomson-Houston Company and the electrical contractors, Messrs. Donnison and Sillem, they have produced results which are well worth the attention of hospital committees and boards.

The general illumination of the wards is provided by a specially designed fitting of the "Eye-Rest" lamps of the Thomson-Houston Company. The lamps are contained in bowls, which contain silvered-glass reflectors; these direct the light from the filaments on to the ceiling, whence it is irradiated downwards as a diffused mellow light altogether free from glare. Bowl and reflector can easily be removed together for cleaning. All the fittings and decorations of the wards are white, which assists this method of illumination very materially.

For local lighting, an enamelled bracket is placed over each bed, equipped with a metal filament lamp. An opaque "Mazdalux" reflector, covered outside with porcelain enamel, keeps all direct rays

Ward Illuminated by Reflected Light.

from the patient's face, nor do the patients in neighbouring or opposite beds perceive any glare from the source of light. Thus one bed may be thoroughly illuminated for any necessary purpose,

An Ingenious Method of Theatre Lighting.

without any general illumination of the ward sufficient to interfere with the comfort of other patients.

In the operating theatre, too, there are ingenious novelties in the (artificial) lighting arrangements. Twenty-four prismatic glass reflectors, each containing a 40-watt lamp, are fixed at a height of ten feet above the surface of the operating table. To avoid any risk of dust dropping off them on to the field of operation, the whole equipment is covered on the underside with a sheet of polished wired plate glass, flush with the ceiling, and on the outside by a water-tight casing. A further advantage of this system is that the engineer-in-charge can get at the lighting equipment without ever entering the theatre at all. The illumination thus afforded is excellent, though experience has yet to show how it compares with less elaborate methods from the point of view of economy.

## Figures and Tables

**Figure f1:**
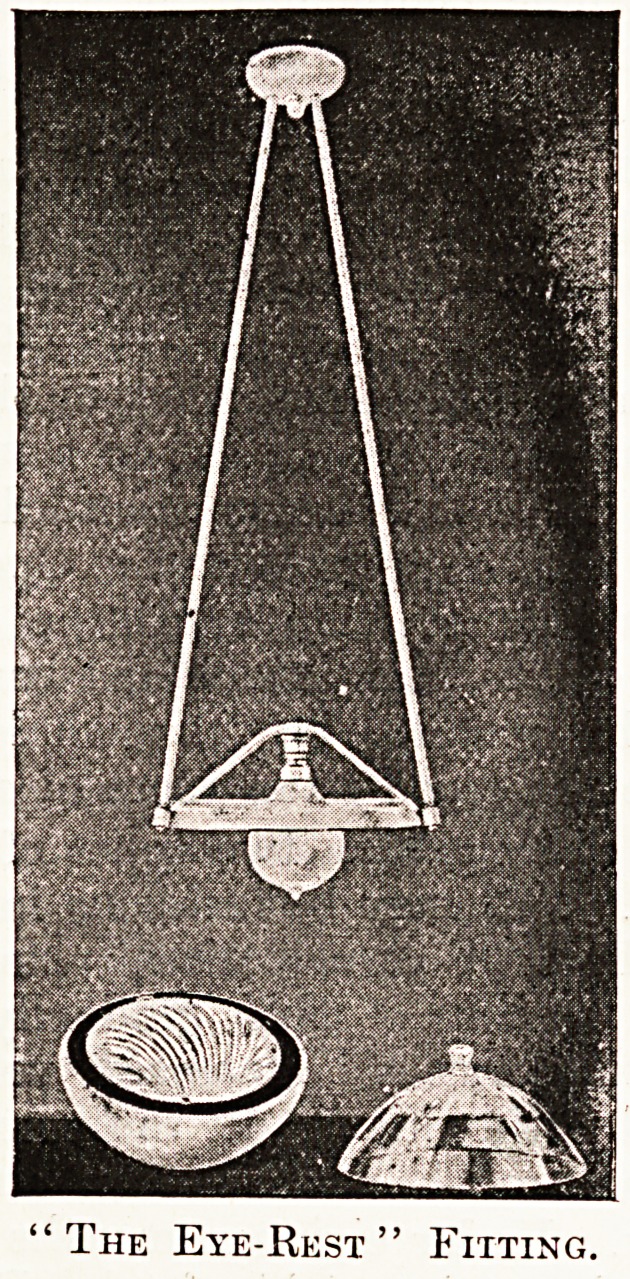


**Figure f2:**
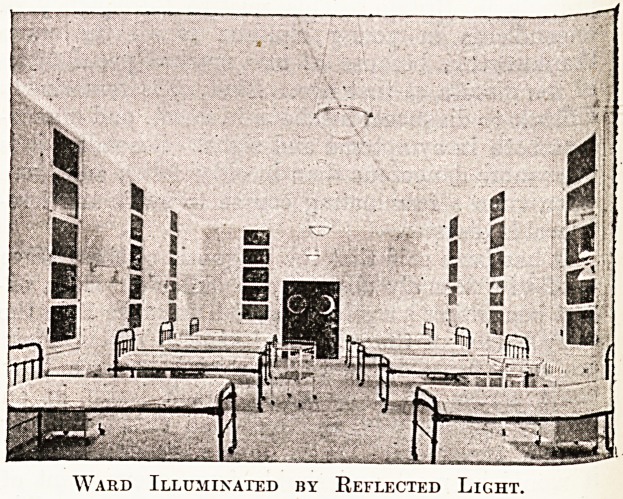


**Figure f3:**